# Sensor Fusion Approach for Multiple Human Motion Detection for Indoor Surveillance Use-Case

**DOI:** 10.3390/s23083993

**Published:** 2023-04-14

**Authors:** Ali Abbasi, Sandro Queirós, Nuno M. C. da Costa, Jaime C. Fonseca, João Borges

**Affiliations:** 1 Algorithmic Center, University of Minho, 4800-058 Azurém, Portugal; 2 School of Medicine, University of Minho, 4710-057 Gualtar, Portugal; 3 2Ai-School of Technology, IPCA, 4750-810 Barcelos, Portugal

**Keywords:** neuromorphic vision sensor, multiple human motion detection and tracking, multi-modal data, sensor fusion, indoor surveillance, event-based data

## Abstract

Multi-human detection and tracking in indoor surveillance is a challenging task due to various factors such as occlusions, illumination changes, and complex human-human and human-object interactions. In this study, we address these challenges by exploring the benefits of a low-level sensor fusion approach that combines grayscale and neuromorphic vision sensor (NVS) data. We first generate a custom dataset using an NVS camera in an indoor environment. We then conduct a comprehensive study by experimenting with different image features and deep learning networks, followed by a multi-input fusion strategy to optimize our experiments with respect to overfitting. Our primary goal is to determine the best input feature types for multi-human motion detection using statistical analysis. We find that there is a significant difference between the input features of optimized backbones, with the best strategy depending on the amount of available data. Specifically, under a low-data regime, event-based frames seem to be the preferred input feature type, while higher data availability benefits the combined use of grayscale and optical flow features. Our results demonstrate the potential of sensor fusion and deep learning techniques for multi-human tracking in indoor surveillance, although it is acknowledged that further studies are needed to confirm our findings.

## 1. Introduction

Security in indoor environments (such as shopping malls, restaurants, offices, or houses) has always been a significant concern, with activities including abnormal event detection, congestion analysis, person identification, and violent action recognition [1,2]. Indeed, having a higher level of security in urban areas has become one of the most important factors in our daily lives. Similarly important, securing a house and working environment with the purpose of preventing any kind of theft or robbery has become more imperative than in the past. Available surveillance systems are often manually controlled and require human supervision, which is a tedious and time-consuming task, with their efficacy prone to being affected by human mistakes and neglects [3,4]. In this sense, automating this surveillance task by tracking people and/or recognizing any noticeable, unwanted, or illegal activity/object appears to be crucial for improving security, deterring criminals with alarms, or calling security forces promptly. Recent advancements in computer vision, namely with the aid of deep learning algorithms, are dramatically changing the trend of video surveillance. Despite the great deal of work done in the past decades on human action recognition with computer vision algorithms, recent state-of-the-art deep learning algorithms and advanced cameras have created a huge opportunity towards improving intelligent surveillance systems, increasing their applicability, and making them more efficient and accurate [3].

Surveillance systems typically use conventional RGB cameras to capture the outside scene and, less often, use infrared cameras for night vision inspection. However, for the task of multiple targets’ detection and tracking, it is crucial to lower the detection error probability and increase reliability. The use of multimodal data is a potential solution [5]. The most common drawback associated with a RGB sensor in connection with object motion detection is that it does not provide raw information concerning moving objects. A good alternative is to use optical flow, which intrinsically contains information about the motion of the objects in the scene while being unaffected by background changes [6,7,8,9,10]. Another alternative sensor is the neuromorphic vision sensor (NVS), which is a newer type of sensor whose capabilities for surveillance applications have yet to be well investigated. NVS sensors are primarily used in object tracking, environment monitoring, and gesture recognition, specifically in scenarios in which objects are moving rapidly and causing blurred images in conventional RGB sensors. NVS sensors are named event-based cameras and are a kind of data-driven sensor whose output depends on the amount of motion and brightness change in the scene, with every pixel responding asynchronously and independently. The faster the motion, the more events per second are generated due to the changing of the sensor’s sampling rate with respect to the rate of change in intensity signal it receives. With NVS sensors, the output is not a frame but a stream of asynchronous digital events. The speed of streaming is not limited by any traditional concept such as exposure time or frame rate. It can detect fast motion with a very low latency, which would traditionally be captured only using expensive, high-speed cameras running at thousands of frames per second, but drastically reducing the amount of data. Considering the advantages and limitations of each sensor, sensor fusion presents itself as a key approach towards improving current surveillance systems. This study aims to find out the effect of grayscale, optical flow, and event-based frames (E-F) input datatypes followed by early sensor fusion of the mentioned input datatypes in multiple object detection (using CNN-based backbones) and tracking on a low-resolution indoor surveillance dataset. The rest of this paper is structured as follows: in Section 2 and Section 3, we introduce the related works and the methodology used in this work, respectively. Section 4 serves as a statistical analysis, followed by experimental results presented in Section 5. Section 6 deals with discussion regarding our results. Finally, we conclude this paper in Section 7.

## 2. Related Works

A specific type of video surveillance system involves the detection and tracking of people and other objects in public or private scenes using cameras. Formerly, the function of a basic video surveillance system was to acquire data from one or many cameras by streaming them to a central location for recording or monitoring. Nowadays, video surveillance systems are equipped with online data analysis systems that can be implemented to ensure security [11,12]. 

There are two main approaches to be selected to deal with multiple-object detection problems, namely, two-stage and single-shot approaches. In the two-stage approach, first the proposed regions are extracted, and then in the next stage, the object detection and classification task will be done. The most well-known two-stage detectors include region convolutional network (RCNN) [13], Fast-RCNN [14], Faster-RCNN [15], Mask-RCNN [16], and FPN [17]. However, the one-shot detectors predict bounding boxes over images without requiring a region proposal step, thereby increasing the detection speed. 

A variety of one-shot methods have been proposed for object detection, including Yolo [18], Yolovx (x = 2:5) [11,19,20], SSD [21], DSSD [22], Resnet [23], RetinaNet [24], and RefineNet [25]. Yolo (You Only Look Once) is a popular real-time object detection system that uses a single neural network to predict bounding boxes and class probabilities directly from full images. Yolovx (You Only Look Once version x) is an extension of Yolo that improves upon its accuracy and speed, with different versions (x = 2:5) achieving different levels of performance. SSD (single shot detector) is another popular one-shot method that uses a single network to simultaneously predict object categories and their locations. DSSD (deconvolutional single-shot detector) extends SSD by adding a deconvolutional module to recover spatial information. Resnet (residual network) is a deep convolutional neural network that uses residual connections to improve performance on very deep networks. RetinaNet is a one-stage detector that uses a feature pyramid network to handle objects at different scales. RefineNet is a multi-path refinement network that iteratively improves the segmentation of objects. Despite their differences, all these methods aim to detect objects accurately and efficiently in images and/or videos, making them important tools in computer vision applications.

**Figure 1:**
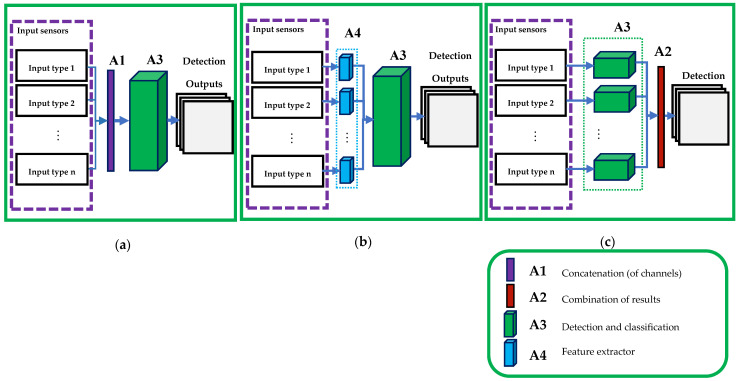
Sensor fusion approaches. (**a**) Low-level data fusion that concatenates/combines different raw input features and feeds them to a single detector. (**b**) Medium-level sensor fusion that fuses coherent features extracted by independent extractors and feeds such data to a detector. (**c**) High-level sensor fusion, in which raw data inputs are fed into individual detectors and the attained results combined in a probabilistic or deterministic way to reach the final detection result.

Sensor fusion approaches for detection-based multiple object tracking (MOT) can be generally categorized into four main types (Figure 1): low-level (early fusion), medium-level (mid fusion), high level (late fusion), and multiple-level fusion. The early sensor fusion (a) incorporates the same or different sensor types as cooperative or complementary data to be fed into the detection network. The medium-level sensor fusion (b) uses a feature extractor as a medium layer to prepare appropriate data for the main detector network. Regarding the late fusion paradigm (c), it uses the high-level data obtained by multiple detectors and only fuses the decisions later.

## 3. Methodology

This section introduces multiple objects tracking algorithms and subsequently presents the early fusion multi-modal architecture for multi-object detection and tracking by utilizing grayscale, optical flow, and event-based frames. Then, we introduce our indoor surveillance multi-human detection dataset. We also provide a detailed ablation study to analyze the gains of multi-sensor fusion by performing experiments on our proposed dataset. 

This study explores the capabilities of low-level sensor fusion in the context of multiple human detection and tracking. The multiple-object motion tracking approach selected for this study is an online (one-shot) detection-based methodology based on FairMOT [26], consisting of two main phases: the first responsible for the detection of multiple objects in the scene using conventional or convolutional methods; and the second employing tracking algorithms to track the detected objects over subsequent frames. The selected tracking methods are intersection-of-union (IoU), re-identification embedding features, and the Kalman filter. The way that the algorithm executes the tracking task is by assigning the most relevant bounding box through the Hungarian assignment method. In this type of approach, the tracking accuracy is highly dependent on the accuracy of the detection module. 

**Figure 2:**
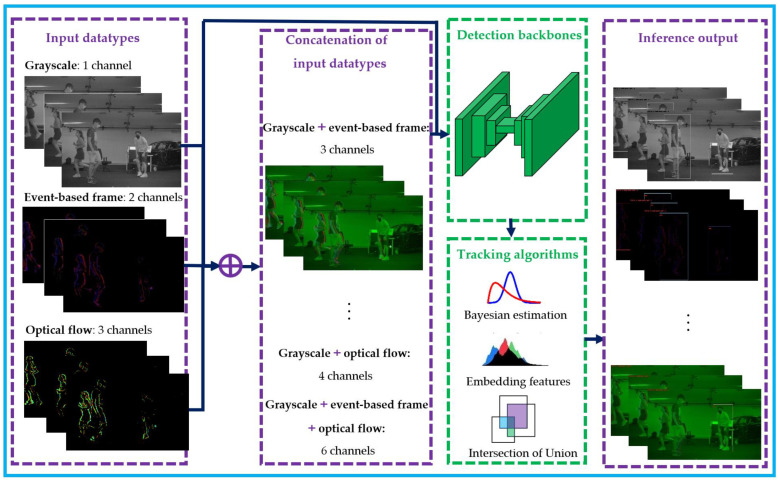
Main pipeline for the early sensor fusion approach.

Our main methodology in this study involves using an early feature fusion approach to address the challenges of multi-human detection and tracking in indoor surveillance. Specifically, we explore the potential benefits of using a low-level sensor fusion approach that combines grayscale, optical flow, and event-based frames obtained from a neuromorphic vision sensor (NVS). To do so, we concatenate the different input feature types channel-wise and feed them into a detector layer, which is a deep convolutional network configured to perform bounding box detection. After the detection phase, the resulting bounding boxes are then passed to various tracking methods, including Bayesian estimation, embedding features, and intersection of unions, for predictions, comparisons, and bounding box assignment tasks. The early sensor fusion pipeline is depicted in Figure 2, which illustrates how the different input features are concatenated and fed into the detector layer. Our approach provides a way to address the challenges of multi-human detection and tracking in cluttered indoor environments, where occlusions, illumination changes, and human-human and human-object interactions can hamper the tracking process. By combining different input feature types, we aim to improve the accuracy and robustness of the detector, particularly in cases where data is limited.

### 3.1. Dataset Creation

We have compiled an indoor dataset consisting of 18 scenarios with a total length of 6973 frames recorded at 15 frames per second and a total of 16,563 bounding boxes. The total number of subjects used in the recordings and the average number of persons appearing in each frame are 12 and 3.5, respectively. The dataset comprises subjects performing different actions (i.e., walking, jogging, sitting, handshaking, shooting, or hugging). Given the limited space imaged and the number of subjects moving, occlusions are frequent across these scenarios, which may hinder the accurate detection and re-identification of each subject.

The Celepixel-V sensor (CelePixel Technology Co., Ltd., Shanghai, China) with a resolution of 1280 × 800 pixels was used during the acquisition. This sensor possesses two functioning modes: the fixed mode and the loop mode. The former outputs pure event-based data, while the latter outputs three different output types (grayscale, optical-flow, and event-based data) in a sequential order. Heretofore, we employed the loop mode, configuring the acquisition duration of each modality.

The event-based data format given by the sensor is converted from bin to csv files containing triggered pixel coordinates, intensity related to amount of brightness change, timestamp, and pixel polarity. The event-based data are then preprocessed and converted to event frames by aggregating the events during a certain event period and putting them in two channels representing positive and negative polarity. Then, each channel is normalized by its maximal value. 

In this work, we expand our dataset to contain additional input data types by concatenating the intrinsic data (grayscale, optical flow, and event-frames) together. The first manipulated input data type is created by concatenating the grayscale and event-based data, resulting in a 3-channel input. Similarly, we concatenate the grayscale data with the optical-flow data, resulting in 4 channels. Finally, all three intrinsic data types are concatenated, resulting in a 6-channel input. The aim of this dataset expansion is to provide a good floor for data-fusion study on the available intrinsic data types. In total, we have 3 datasets created with basic datatypes and 3 more datasets generated by combining the originally acquired datatypes. 

### 3.2. Dataset Preparation

**Figure 3:**
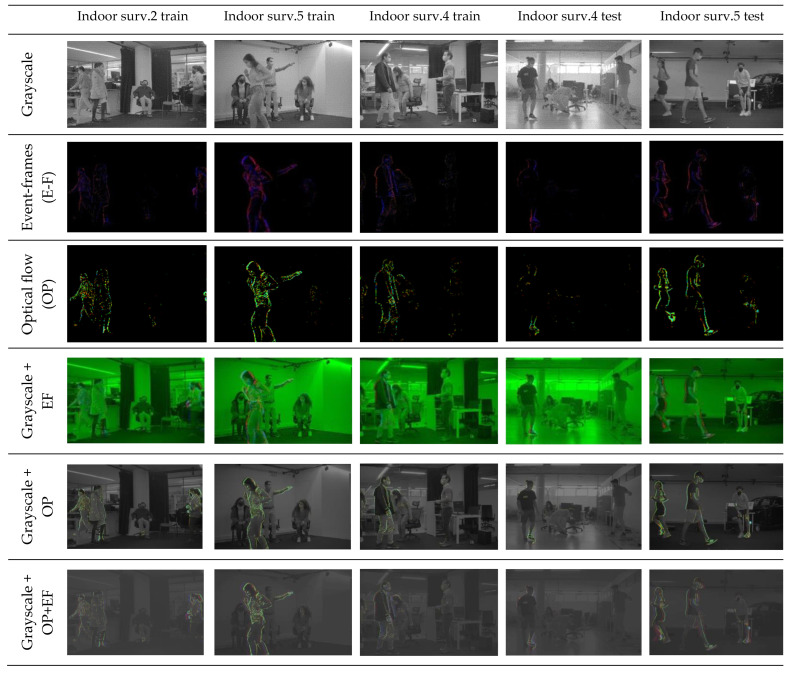
Sample frames of each acquired input in five scenarios.

The present dataset was carefully annotated with ground truth bounding boxes drawn around each human over the full video segments, ensuring that our models were trained on accurate and reliable data. To ensure that our experimental results were both robust and generalizable, we curated a diverse dataset consisting of 18 different scenarios, each recorded in a different indoor environment. To increase variability, scenarios with varying levels of illumination, clothing types, colors, and activities, such as walking, running, and standing still, were captured. For each scenario, we recorded data from multiple modalities, including grayscale, optical flow, and event-based frames with an NVS camera. To avoid overfitting to specific scenarios, we split the dataset into training, validation, and test sets, with 8, 4, and 6 scenarios, respectively. Figure 3 provides a visual representation of five of the scenarios in our dataset. It is worth noting that each scenario was recorded in a different indoor space with different people, making the dataset highly diverse. Although the dataset was relatively small, the diversity of scenarios allowed us to train models that generalize well to new and unknown scenarios. To further mitigate overfitting, we employed techniques such as data augmentation, regularization, and dropout during training.

### 3.3. Experimental Design

The objective of the study is to carry out different experiments to explore the sensor datatypes capabilities and investigate the effect of early sensor fusion on human detection and tracking scores on a low-regime dataset. To achieve this, six different sets of datatypes (3 original and 3 fused) were fed to four distinct multi-object detection backbones. The selected backbones are pose-dla-dcn-34, resnet-dcn-34, resnet-dcn-50, and resnet-fpn-dcn-34.

To make the description of further experiments and their associated results simpler, Table 1 summarizes the variants tested and their respective symbolic names.

**Figure 4:**
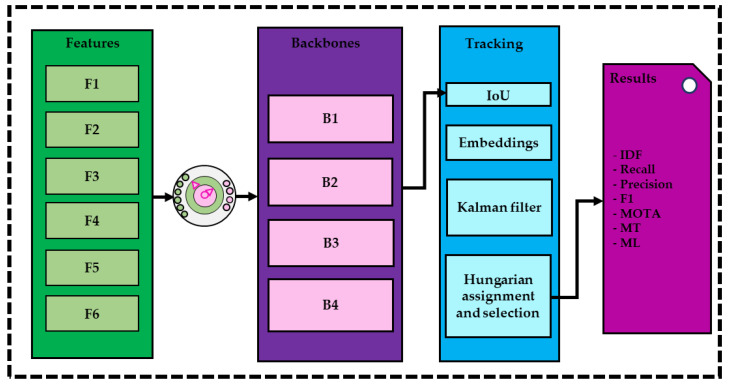
Experiments with different input features and different backbones with considering the tracking methods.

All networks were trained for 30 epochs with a batch size of 12. Two types of augmentation were used: spatial augmentation and brightness augmentation. The spatial augmentation included affine transformations and was applied to all datatypes. Given the impossibility to apply brightness augmentation in event-based and optical flow frames, it was just applied to the grayscale input type (or respective channel when using early sensor fusion). Figure 4 shows the graphical experimental design used in our study.

### 3.4. Hardware

The experiments were performed on a server with a CPU Intel(R) Xenon(R) Gold 6140 @ 2.3 GHz and two GPUs (a Tesla V100 PCIE 32 GB and a Tesla V100 PCIE 16 GB), with a total of 100 GB of RAM.

### 3.5. Optimization

Given the limited size of our dataset, the training of the above architectures is highly susceptible to overfitting. Moreover, the susceptibility to overfitting also depends on the input feature employed. Thus, to reduce the effects of overfitting and improve the results, a few initial experiments were performed, modifying hyperparameters such as L2 regularization and the use (or not) of brightness augmentation. Table 2 shows the range of values used for optimizing the results of each input feature. 

Table 3 summarizes the values that led to the best performance per input-type.

**Figure 5:**
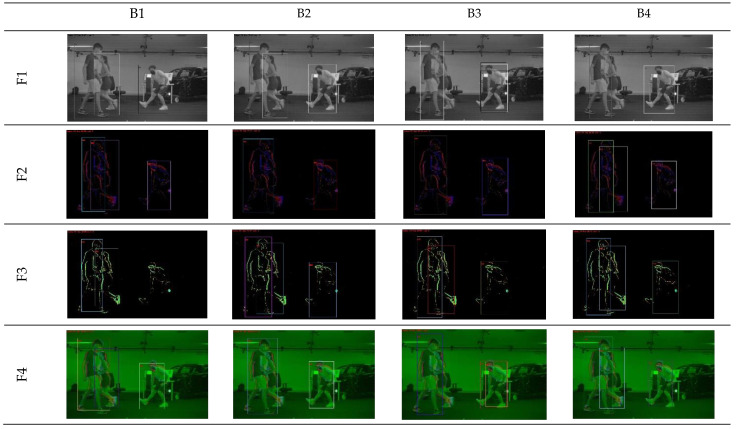
Detection results achieved by 4 different backbones and 4 selected input types.

Figure 5 illustrates some detection results achieved with distinct input datatypes and backbones.

## 4. Statistical Analysis

A nonparametric unpaired *t*-test (Mann–Whitney test) was carried out to compare the F1-scores achieved by optimized and non-optimized models. Significance was assumed if ρ < 0.05 (two-tailed). Moreover, the same test was performed to examine whether there is any significant difference between the F1-scores of distinct backbones in the optimized vs. non-optimized models. Similarly, nonparametric Dunn’s multiple comparison tests were conducted to statistically compare the F1-scores between input datatypes. A similar analysis was performed to compare the F1-scores between backbones. 

## 5. Experimental Results

**Figure 6:**
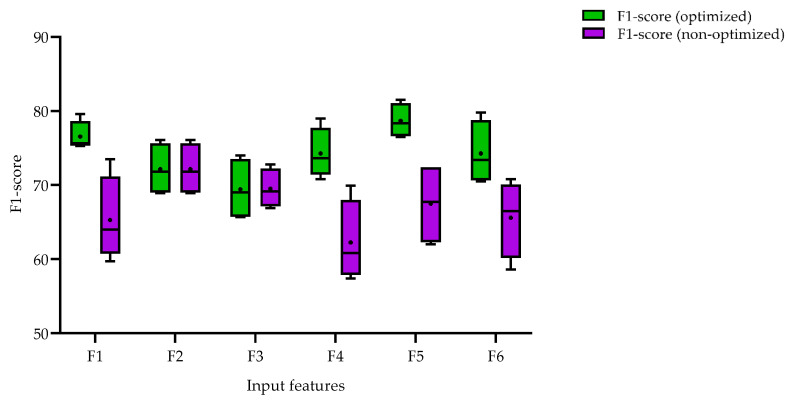
F1-score results obtained by optimized and non-optimized trainings, when divided by input datatype.

In this study, we conducted experiments to evaluate the performance of multi-human detection using two different approaches, as shown in Figure 6. The first approach involved conducting experiments without optimization in training the input features against different backbones, while the second approach used optimizations for all experiments. For each of the two approaches, we experimented with several input data types (F1, F2, …, F6) against various backbones and calculated the F1-score for each experiment. The purpose of defining these two approaches was to explore the most efficient input features without running extensive and time-consuming experiments for hyperparameter optimization, as well as to avoid overfitting issues. We observed that, for the non-optimized scenario, the event-based frame input data type has, on average, a higher F1-score compared to other input data types. Interestingly, the average F1-score obtained from the event-based frame input data type in the non-optimized scenario was the same as the score obtained from the optimized scenario. Our findings suggest that incorporating optimization into the training process can improve overall detection performance. Furthermore, we observed that certain input features, such as grayscale and combined features, performed better on average than others. Overall, our results provide insight into the impact of input data types on multi-human detection performance.

### Input Datatype Wise Comparisons

The Mann–Whitney U test results associated with each of the input data types are shown in Table 4. Significant differences (*p*-value < 0.05) were found between optimized and non-optimized backbones for F1, F4, and F5, but not for F2, F3, and F6 (although only marginally for the latter). Whereas we are dealing with a small population, we expect to get false positive results from the test, so we emphasize the q-value, which adjusts the p-value for finding the false discovery rate (FDR). The calculated *q*-values, point out a narrow rejection of the null hypothesis (there is a significant difference between optimized and non-optimized backbones for each input datatype). Accentuating non-parametric test results obtained by the Mann–Whitney U test, the same results are obtained by comparing the *p*-values. 

For studying all possibilities, nonparametric one-way ANOVA tests (Kruskal–Willis tests) were performed to compare the F1-score median differences of each input datatype versus other input datatypes, both for optimized and non-optimized models. The overall obtained *p*-values associated with optimized and non-optimized models are 0.0385 and 0.0626, and the Kruskal–Willis statistics are 11.75 and 10.48, respectively. Therefore, these values overall show that there are significant differences between the F1-score of all input features versus other input features. Table 5 shows the individual statistical results of each input feature vs. other input features using Dunn’s multiple comparison test (the upper green triangle and lower violet triangle matrix relate to optimized and non-optimized models, respectively). As it is shown, only F5 vs. F3 (optimized model) and F4 vs. F2 (non-optimized model) have significant differences with each other, while for others the null hypothesis is rejected and retained. 

## 6. Discussion

Our experiments indicate that, in a non-optimized scenario, the event-based input datatype outperforms other single or combined features in terms of detection accuracy. We hypothesize that such a result is justified by the limited size of our dataset combined with the fact that grayscale and optical flow features are more complex to learn (notice the textures present in the grayscale frames), making them more susceptible to poor generalization. In fact, when using event-based data alone, the use of L2 regularization did not result in any performance improvement. Contrarily, for the other input datatypes, it was essential to experiment with distinct amounts of data augmentation and regularization, especially when employing grayscale features (again, due to their texturized appearance). 

Regarding the results for the optimized models, our results highlight F5 (grayscale plus optical flow) as providing a noteworthy gain in detection accuracy. This particular result should not imply that other concatenated input datatypes (F4 and F6) are inferior to F5 in achieving higher scores. Furthermore, those differences could be related to the optimization process and the dataset size, increasing the likelihood of overfitting when training with a more complex fused input datatype when compared to one of the basic datatypes. Indeed, the more complex the input feature, the harder it was to perform optimization to minimize overfitting for such small data. 

According to the results derived from non-optimized experiments, it indicates that for a low dataset regime of training, if either there is a lack of sufficient hardware resources or a shortage of time for a long-term training’s optimization journey, an event-based frame dataset can be a good candidate since it is immune from any overfitting and does not require optimization during training.

## 7. Conclusions

In conclusion, this nonparametric experimental study investigated early data fusion methods using grayscale, optical flow, and event-based frames acquired by an NVS sensor. The study results indicate that event-based frames are the preferred input feature type for non-optimized approaches, while the combination of grayscale and optical flow input data types appears to perform better for optimized approaches. Moreover, the study’s significant findings demonstrate that optimization for small regime data and limited combinations of experiments work well for grayscale and combined input features, while for event-based frames, there was no significant difference. This may lead to new applications of event-based frames in scenarios where heavy optimization for training is not feasible. Overall, this study contributes to the existing literature on early data fusion methods using NVS sensors and has potential implications for practical applications in the field. However, the findings are limited by the small size of our dataset and nonparametric tests, which have low statistical power. Further research is required to confirm our hypotheses, including exploring larger datasets and comparing different input feature types and fusion methods against a larger number of multi-object detection algorithms with different backbones and structures.

## Figures and Tables

**Table 1:** Symbolic naming of input features and backbones.

Input Features	Naming’s	Backbones	Naming’s
Grayscale	F1		
Event-based frame (E-F)	F2	Pose-dla-dcn-34	B1
Optical flow	F3	Resnet-dcn-34	B2
Grayscale + E-F	F4	Resnet-dcn-50	B3
Grayscale + optical flow	F5	Resnet-fpn-dcn-34	B4
Grayscale + optical flow + E-F	F6		

**Table 2:** Optimization parameters selected to reduce overfitting.

Regularization	Brightness Augmentation	Noise
1 × 10^−1^ to 1 × 10^−6^	Multiplication method Uniform distribution σ = 0.1 and mean = 0.1	Adding method Uniform distribution σ = 0.1 and mean = 0.1

**Table 3:** Best values of augmentation per input-type used.

Input Features	Regularization	Brightness Augmentation
F1	1 × 10^−2^	✓
F2	-	-
F3	1 × 10^−4^	-
F4	1 × 10^−2^	Applied on grayscale channel
F5	1 × 10^−2^	Applied on grayscale channel
F6	1 × 10^−2^	Applied on grayscale channel

**Table 4:** Optimized vs non-optimized unpaired t-test (non-parametric test).

Feature	*p*-Value	Mann–Whitney U	*q*-Value
F1	0.0285	0.001	0.057
F2	0.999	8.000	0.999
F3	0.886	7.000	0.999
F4	0.0285	0.001	0.0577
F5	0.0285	0.001	0.0577
F6	0.0571	1.000	0.086

**Table 5:** Kruskal-Wallis test for feature mean comparison of optimized backbones.

Kruskal–Wallis Test			Optimized Models		Non-Optimized Models	
***p*-value**			0.0385		0.0626	
**Number of groups**			6		6	
**Alpha**			0.05		0.05	
**Kruskal–Wallis statistics**			11.75		10.48	
**Dunn’s multiple comparison test**						
**Features**	**F1**	**F2**	**F3**	**F4**	**F5**	**F6**
**F1**		>0.999	0.502	>0.999	>0.999	>0.999
**F2**	0.535		>0.999	>0.999	0.268	>0.999
**F3**	>0.999	>0.999		>0.999	0.028	>0.999
**F4**	>0.999	0.034	>0.999		0.859	>0.999
**F5**	>0.999	>0.999	>0.999	>0.999		>0.999
**F6**	>0.999	0.416	>0.999	>0.999	>0.999	

## Data Availability

Not applicable.
